# Assessment of Visual Evoked Potential to Detect Visual Pathway Dysfunction in Gestational Diabetes Mellitus: A Longitudinal Case-Control Study With Postpartum Follow-up

**DOI:** 10.7759/cureus.49619

**Published:** 2023-11-29

**Authors:** Akriti Kapila Sharma, Latika Mohan, Sunita Mittal, Anupama Bahadur, Anissa A Mirza, Manish Thapiyal

**Affiliations:** 1 Physiology, All India Institute of Medical Sciences, Rishikesh, Rishikesh, IND; 2 Obstetrics and Gynecology, All India Institute of Medical Sciences, Rishikesh, Rishikesh, IND; 3 Biochemistry, All India Institute of Medical Sciences, Rishikesh, Rishikesh, IND

**Keywords:** longitudinal study, visual pathway dysfunction, diabetes in pregancy, visual evoked potentials, gestational diabetes mellitus

## Abstract

Background: The increasing prevalence of gestational diabetes mellitus (GDM) during pregnancy has opened the opportunity to study its short- and long-term effects on maternal ophthalmic health. Visual evoked potential (VEP) is a non-invasive electrophysiological test that detects functional disturbances along the visual pathway before the physical signs of diabetic retinopathy (DR) can set in. This longitudinal study is aimed at the assessment of changes in VEP in GDM during different stages of pregnancy and 6-12 weeks after parturition by comparing it with normoglycemic controls.

Design and method: Diagnosed cases of GDM were recruited along with normoglycemic controls at 24-28 weeks of gestation. Each participant was required to attend two follow-up appointments at 32-38 weeks of gestation and 6-12 weeks after parturition. A blood sample was taken in a fasting state to record biochemical parameters. VEP was recorded using Neuropack S1 MEB-9400 electrodiagnostic equipment (Nihon Kohden, Tokyo, Japan) in a dark room by providing pattern reversal stimuli to each eye.

Results: A total of 29 participants (15 in the control group and 14 in the GDM group) completed the entire study procedure. The observed mean P100 latency of both eyes in the GDM group was recorded longer as compared to that in the control group at baseline and during late pregnancy. Although the mean P100 latency saw a significant decline in postpartum visits as compared to that in late pregnancy, the values were higher than in the control group. P100 latency at baseline correlated significantly to serum advanced glycated end products' (AGE’S) levels in the GDM group.

Conclusion: Our study findings reflect that the diagnosis of GDM is associated with significant changes in VEP during and after pregnancy as compared to that of healthy pregnant women.

## Introduction

Gestational diabetes mellitus (GDM) is a form of disruption in carbohydrate metabolism that is detected for the first time during pregnancy causing glucose intolerance [1]. Diabetes in pregnancy (DIP) is the most common antenatal complication, affecting one in six pregnancies worldwide, and GDM contributes to 80% of cases of DIP [2]. Although GDM is known to regress after parturition, it can lead to a trail of short- and long-term complications for both mother and offspring [3], including a risk of post-partum type 2 diabetes mellitus (DM) in 20% of affected women [4]. The increasing prevalence of GDM during pregnancy has led clinicians and researchers to focus on its short- and long-term effects on maternal ophthalmic health, including a potential risk for future diabetic retinopathy (DR) [5]. A population-based study by Beharier et al., exploring the association of post-partum ophthalmic morbidity with GDM, found that patients diagnosed with GDM had an increased incidence of DR, along with glaucoma and retinal detachment disorder during a 12-year follow-up [5].

Visual evoked potential (VEP) is a non-invasive electrophysiological test that evaluates the functioning of visual pathways [6]. VEP detects functional changes in the retinal pre-ganglionic and ganglionic cells, as well as delayed conduction along the visual pathway that occurs before the onset of retinal microvascular damage characteristic of DR [7]. Such functional changes can occur due to the shift in the metabolic environment characteristic of diabetes [7]. Alterations in VEP have been reported as early as less than a month after diagnosis of type 1 and type 2 DM in children [8] and less than six months after diagnosis of type 1 DM in adults [9,10]. Short-term fluctuations in glycaemic status of type 2 DM cases have been observed to cause alterations in VEP [11-13].

Documentation of VEP changes associated with GDM during and after pregnancy is limited in the literature. A pilot study by Kumar et al. found significantly prolonged P100 latency in the VEP test in the GDM population as compared to normoglycemic pregnant women [14]. There was a direct correlation of P100 latency with the level of hyperglycemia in GDM. This study was cross-sectional and lacked post-partum follow-up [14]. We could not find any other study documenting changes in VEP associated with GDM. With the growing burden of GDM, VEP can provide a non-invasive tool to identify at-risk GDM patients for follow-up and monitoring for DR [14].

Our study was aimed at a longitudinal assessment of changes in pattern-reversal VEP using Neuropack S1 MEB-9400 electrodiagnostic equipment (Nihon Kohden, Tokyo, Japan) in subjects with GDM during different stages (24-28 weeks and 32-28 weeks of gestation) of pregnancy and 6-12 weeks after parturition by comparing it with normoglycemic controls. We have attempted to correlate changes in VEP with biochemical markers related to the progression of GDM.

## Materials and methods

This was a longitudinal case-control study conducted in a tertiary care hospital in Northern India. Participants recruited in this study were pregnant women with diagnosed GDM and normoglycemic pregnant women in 24-28 weeks of gestation. The study was approved by the Institutional Ethics Committee, AIIMS, Rishikesh, India.

Recruitment and follow-up of participants

The diagnostic criteria for GDM used in this study was the International Association of Diabetes in Pregnancy Study Group (IADPSG) guidelines, 2008, based on fasting 75-g oral glucose tolerance test (OGTT) [15]. Pregnant women with hypertension in pregnancy, cardiovascular diseases, a history of central nervous system (CNS) injury, and ophthalmic lesions were excluded from the study. Pregnant women with overt diabetes (ODM) and pre-gestational diabetes were also excluded from the study.

Recruitment of the participants was done by convenience sampling from the Outpatient Department (OPD) and ward of the Department of Obstetrics & Gynaecology. Written informed consent was obtained from the participant before starting the study procedure. Each participant was supposed to attend two follow-up appointments scheduled at 32-28 weeks of gestation and 6-12 weeks after parturition. Out of 66 participants inducted in the study, 50 reported for the first follow-up appointment, and 29 reported for the second follow-up appointment. Reminders through calls and text messages were sent to each participant to report for the scheduled appointment.

Data collection

Subjects screened and willing to participate in the study were called early morning in a fasting state. Specifically, 10 ml of venous blood was collected for assessing the baseline biochemical parameters, including fasting plasma glucose (FPG) estimated using a hexokinase enzymatic colorimetric method on a fully automated analyzer (Beckman AU 680; Beckman Coulter Diagnostics, Brea, California). Fasting insulin was estimated using chemiluminescent immunoassay (CLIA) with a commercially available kit (Insulin CLIA kit; Cortez Diagnostics, Inc., Woodland Hills, CA). Glycated hemoglobin (HbA1C) was estimated using high-performance liquid chromatography (HPLC) (HLC, Automated glycohemoglobin analyzer; Tosoh India Pvt. Ltd., Mumbai). Finally, serum N-carboxymethyl lysine (N-CML), a form of advanced glycated end product (AGE), was analyzed using competitive inhibition- enzyme-linked immunosorbent assay (ELISA) technique using a commercially available kit (ELK Biotechnology Co. Ltd., China, Catalogue: ELK7896).

After a light breakfast, anthropometric parameters, including weight, height, BMI, and head circumference of the subjects, were measured. VEP was measured in the Electrophysiology Laboratory, Department of Physiology. Anthropometric and biochemical variables such as HbA1C, fasting insulin, and N-CML were measured during the first visit and post-partum follow-up, while VEP and FPG were recorded for all three appointments. Retinal screening through ophthalmoscopy was done for GDM women during the third trimester of pregnancy and 6-12 weeks after parturition to ascertain DR. None of the GDM cases included in the study reported any physical signs of DR during antenatal and postnatal ophthalmoscopic screening.

The VEP was recorded in a dark room by using pattern reversal stimulation of each eye. Pure silver cup electrodes were placed as per the International 10-20 System of EEG electrode placement. The active electrodes were placed 5 cm left and right to Oz, namely, Lo and Ro, respectively. The ground electrode was placed at Cz, and the reference electrode was placed at Fz. Interelectrode impedance was kept below 5 Ohms. A total of 200 stimulations were provided to each eye to obtain two waves - Lo-Fz and Ro-Fz. The wave Lo-Fz of the left and right eyes was used for statistical comparison. The latencies and amplitude of N75 and P100 waves were marked for comparison. The Lo-Fz wave of the left eye of both groups was used for correlation and regression analysis. ISCEV guidelines were followed for the calibration of equipment and procedures [16].

Statistical analysis

Statistical analysis was done using Stata software (StataCorp LLC, College Station, Texas). Continuous data were represented as mean with standard deviation or median with an interquartile range as appropriate. The normality of the continuous data was measured using a skewedness-kurtosis test. Outcomes were compared using an independent t-test or the Mann-Whitney U test as appropriate for a single visit between the two groups. Comparison of FPG, P100 latency, and N75-P100 amplitude within the same group for all three visits was done using repeated measures ANOVA or the Friedman test as appropriate. Comparison of BMI, HbA1C, fasting insulin, and N-CML within the same group between the first and third visits was done using paired t-test or Wilcoxon signed rank tests as appropriate. Correlation was performed using Pearson’s or Spearman’s correlation analysis as appropriate followed by multiple linear regression analysis. A p-value ≤ 0.01 was considered statistically significant.

## Results

Study parameters at baseline (24-28 weeks of gestation)

A total of 29 participants (15 in the control group and 14 in the GDM group) completed the entire study procedure. A comparison of demographic, anthropometric, biochemical, and neurophysiological parameters of the control and GDM groups at baseline (24-28 weeks of gestation) is illustrated in Table 1. The two groups were similar in terms of age, gestational age parity, pre-gestational weight, pre-gestational BMI, waist-hip ratio, and head circumference at the time of study. The GDM group reported a family history of diabetes and a history of GDM in previous pregnancy a greater number of times than controls, while the difference in frequency of the latter was statistically significant between both the groups. At baseline, 42.8% of participants in the GDM group were treated with diet therapy, while 57.2% of participants were treated with insulin therapy. Biochemical parameters of FPG, HBA1C, HOMA IR, and N-CML were statistically different between both groups.

**Table 1:** Comparison of demographic, anthropometric, clinical, and biochemical outcomes at baseline (24-28 weeks), late pregnancy (32-38 weeks), and postpartum (6-12 weeks after delivery) between control and gestational diabetes mellitus (GDM) groups. *P-value less than 0.01 is considered statistically significant, ^DM - Diabetes mellitus, @GDM - Gestational diabetes mellitus, ®Initial weight and BMI refer to pre-gestational weight or weight measured in the first antennal visit, as reported in medical records, ιHOMA IR - Homeostatic model of assessment of insulin resistance.

Outcome	Control	GDM	P-Value
Age (in years)	28.4±3.5	28.6±4.9	0.87
Gestational age (in weeks)			
Baseline	27.2±2.8	28±2	0.43
Late pregnancy	33.8±1.9	35.7±2.1	0.02
Postpartum	7.13±2.6	6.4±1.3	0.38
Parity, n (%)			
Nulliparous	4 (26.6)	5 (35.7)	0.59
Multiparous	11 (73.4)	9 (64.3)	
Family history of DM^, n (%)			
Present	4 (26.6)	10 (71.2)	0.01*
Absent	11 (73.4)	4 (28.8)	
History of previous GDM^@^, n (%)			
Present	2 (13.4)	5 (35.7)	0.15
Absent	13 (86.6)	11 (78.5)	
Mode of treatment, n (%)			
Baseline		Insulin therapy 8 (57.2)	
		Diet therapy 6 (42.8)	
Late pregnancy		Insulin therapy 10 (71.4)	
		Diet therapy 4 (28.6)	
Mode of delivery, n (%)			
Lower-segment caesarean section	7 (46.6)	9 (64.3)	0.34
Vaginal delivery	8 (53.4)	5 (35.7)	
Weight (kg)			
Initial weight®	61.29±12.33	65.91±8.57	0.27
Postpartum weight	64.76±12.8	70.6±10.05	0.20
Body mass index (BMI)			
Initial®	23.95±4.01	25.56±4.01	0.31
Postpartum	24.97±4.45	27.24±4.60	0.18
Head circumference (cm)	53.33±2.87	53.5±3.52	0.88
Fasting plasma glucose (mg/dL)			
Baseline	75.41±7.93	96.35±15.51	0.0003*
Late pregnancy	63.13±7.19	86.57±5.41	<0.001*
Postpartum	60.6±4.59	88.28±24.64	0.0002*
Glycated haemoglobin (HbA1C)			
Baseline	3.80±0.68	5.62±0.41	<0.001*
Postpartum	3.4±0.61	4.73±0.98	0.0001*
HOMA IR^ι^			
Baseline	0.92±0.15	1.33±0.22	<0.001*
Postpartum	0.67±0.21	0.96±0.30	0.0086*
Serum advanced glycated end products (AGEs) (ng/dL)			
Baseline	156±33.33	291±17.91	<0.001*
Postpartum	147±40.25	285.6±21.15	<0.001*

The observed mean P100 latency of both eyes of the GDM group was longer, and N75-100 amplitude was smaller in the GDM group than in the control group with the difference being statistically significant. The difference in N75 latency between GDM and control was not statistically significant (Table 2).

**Table 2:** Comparison of VEP between the control and GDM groups at baseline (24-28 weeks of gestation), late pregnancy (32-38 weeks of gestation), and postpartum visit (6-12 weeks after parturition). *P-value less than 0.01 is considered statistically significant, ^GDM - Gestational diabetes mellitus.

Parameter	Control	GDM^	P-value
Baseline recording			
Right eye			
N75 latency	69.42±2.47	70.67±3.72	0.29
P100 latency	97.42±2.24	102.96±3.10	<0.001*
N75-100 amplitude	9.73±1.96	7.27±2.07	0.002*
Left eye			
N75 latency	69.25±2.78	70.17±3.86	0.47
P100 latency	97.65±2.73	102.13±3.22	0.004*
N75-P100 amplitude	7.64±1.69	6.27±0.76	0.009*
Late pregnancy recording			
Right eye			
N 75 latency	69.91±3.04	71.69±2.22	0.09
P100 latency	97.39±2.25	105.52±4.26	<0.001*
N75-P100 amplitude			
Left eye			
N75 latency	74.37±4.25	76.22±4.39	0.25
P100 latency	98.8±1.95	105.07±4.09	<0.001*
N75-P100 amplitude	7.04±2	5.67±0.76	0.226
Postpartum recording			
Right eye			
N75 latency	70.28±2.67	71.03±2.64	0.44
P100 latency	98.05±2.20	100.68±2.53	0.005*
N75-P100 amplitude	8.49±1.67	8.12±2.03	0.59
Left eye			
N75 latency	70.20±2.61	70.35±2.64	0.88
P100 latency	98.29±1.93	100.72±2.40	0.0056*
N75-P100 amplitude	7.93±1.45	7.67±1.39	0.630

Within the GDM group, a comparison of VEP parameters was made between women receiving diet therapy and women receiving insulin therapy. The P100 latency of the left eye was significantly prolonged in the subgroup receiving insulin therapy as compared to the subgroup placed on diet therapy. There was no statistical difference in any other VEP parameter between these two subgroups (Table 3).

**Table 3:** Comparison of VEP parameters between GDM women receiving insulin therapy and GDM women receiving diet therapy at baseline (24-28 weeks of gestation) and late pregnancy (32-28 weeks of gestation). *P-value less than 0.01 is considered statistically significant.

Variable	Diet therapy	Insulin therapy	P-value
Baseline (24-28 weeks of gestation)			
Left eye			
P100 latency (ms)	99.4±2.24	104.16±2.17	0.0019*
N75-P100 amplitude (µV)	6.43±1.02	6.16±0.53	0.531
Right eye			
P100 latency (ms)	101.06±2.87	104.28±2.37	0.04
N75-P100 amplitude (µV)	7.25±1.52	7.61±2.21	0.73
Late pregnancy (32-38 weeks of gestation)			
Left eye			
P100 latency (ms)	104.29±2.91	107.05±6.30	0.270
N75-P100 amplitude (µV)	5.47±0.52	5.75±0.82	0.55
Right eye			
P100 latency (ms)	104.55±3.21	107.25±6.36	0.30
N75-P100 amplitude (µV)	6.83±1.75	7.35±1.65	0.62

Study parameters in late pregnancy (34-38 weeks of gestation) and postpartum follow-up (6-12 weeks after parturition)

In late pregnancy (34-38 weeks of gestation), 28.4% of participants in the GDM group continued being treated by diet therapy, while 72.4% of participants were treated by insulin therapy. The biochemical parameter of FPG was statistically significant between both groups. There was no statistical difference in N75 latency and N75-P100 amplitude of the left and right eyes between the control and GDM groups, while the observed mean P100 latency of both eyes in the GDM group was longer than the control group with the difference being statistically significant (Table 2). Although the mean P100 latency in late pregnancy in the GDM group was longer than the P100 latency recorded at baseline, the difference was not statistically significant (Table 4).

**Table 4:** Comparison of outcomes between the first (baseline at 24-28 weeks of gestation), second (late pregnancy at 32-38 weeks of gestation), and third (6-12 weeks postpartum) visits within the same group. *P-value less than 0.01 is considered statistically significant. ^GDM - Gestational diabetes mellitus.

Group	P-value			
	Overall	1^st^ v/s 2^nd^ visit	2^nd^ v/s 3^rd^ visit	1^st^ v/s 3^rd^ visit
BMI as the outcome variable				
Control	0.003*	----	-----	0.003*
GDM^	<0.001*	-----	------	<0.001*
BA1C as outcome variable				
Control	0.016*	------	-----	0.016*
GDM^	0.013*	------	-----	0.013*
N CML as the outcome variable				
Control	0.270	------	------	0.270
GDM^	0.503	------	------	0.503
P100 L of the left eye as the outcome variable				
Control	0.201	0.230	1.00	0.945
GDM^	0.006*	0.086	0.006*	0.833
N75-P100 A of the left eye as the outcome variable				
Control	0.082	0.283	0.988	0.103
GDM^	<0.001*	0.104	<0.001*	<0.001*

When comparing VEP parameters between women placed on diet therapy and women placed on insulin therapy within the GDM group, there was no statistical difference in the N75 latency and N75-P100 amplitude of the left and right eyes between both subgroups. Although the mean P100 latency of both the left and right eyes was increased for the subgroup receiving insulin therapy, the difference was not statistically significant (Table 3).

In the postpartum visit (6-12 weeks after pregnancy), all our participants reported live birth with a greater number of participants in the GDM group reporting LSCS during childbirth although the difference in the frequency of LSCS during childbirth between GDM and control was not statistically significant. The difference in post-gestational weight and BMI between the GDM and control groups was not statistically significant. Biochemical parameters of FPG, HBA1C, HOMA IR, and - CML were statistically different between both groups (Table 1). VEP of both eyes revealed that there was no statistical difference in the N75 latency and N75-P100 amplitude, while the P100 latency was statistically different for both eyes between the GDM and control groups (Table 2). There was a statistical difference between P100 latency during late pregnancy and postpartum follow-up in the GDM group (Table 4).

Correlation analysis

Correlation analysis revealed that there was a significant positive correlation of P100 latency of the left eye in the GDM group at baseline with the mode of treatment of GDM, HBA1C levels, and N-CML levels (Table 5). There was no other significant correlation of P100 latency during all three visits in the GDM group.

**Table 5:** Correlation of the P100 latency and N75-P100 amplitude of the left eye of the GDM group with anthropometric, obstetric, and biochemical variables during the first, second, and third visits. *P-value less than 0.01 is considered statistically significant, #DM - Diabetes mellitus, ^GDM - Gestational diabetes mellitus, √BMI - Body mass index, ⁓FPG - Fasting plasma glucose, ®HOMA IR - Homeostatic model of assessment of insulin resistance, AGEs - Advanced glycated end products.

Parity		Family h/o DM^#^		History of GDM^		Mode of treatment		BMI^√^		FPG⁓		HBA1C		HOMA IR®		Serum AGEs^ ι^	
r	P	r	P	r	P	r	P	r	P	r	P	r	P	r	P	r	P
With P100 latency as an independent variable																	
1^st^ Visit																	
-0.322	0.26	0.125	1.00	0.05	0.85	-0.84	0.002*	0.41	0.13	0.07	0.78	0.75	0.001*	0.19	0.49	0.80	0.008*
2^nd^ Visit																	
-0.250	0.387	0.319	0.797	0.35	0.21	-0.14	0.62	0.32	0.25	0.12	0.68	0.05	0.85	0.16	0.57	0.37	0.32
3rd visit																	
0.161	0.580	0.195	0.50	0.14	0.61	-0.39	0.16	0.05	0.84	0.01	0.96	0.07	0.81	0.16	0.56	0.41	0.26
With N75-P100 amplitude as an independent variable																	
1^st^ Visit																	
-0.017	0.951	-0.88	1.00	-0.48	0.08	0.10	0.71	-0.01	0.94	0.27	0.33	-0.18	0.53	-0.18	0.52	0.63	0.06
2^nd^ visit																	
0.471	0.088	-0.23	1.00	0.37	0.18	0.01*	0.95	-0.28	0.32	-0.06	0.83	-0.24	0.39	0.01	0.96	0.06	0.86
3^rd^ Visit																	
0.053	0.855	0.10	0.71	0.25	0.37	-0.07	0.80	0.13	0.65	0.60	0.02	0.51	0.06	0.71	0.004*	-0.03	0.91

Regression analysis

Multiple step-wise linear regression analysis was done with the mode of treatment for GDM, HBA1C, and N-CML recorded at baseline as dependent variables and P100 latency of the left eye at baseline as the independent variable in the GDM group. Although HBA1C was a major contributor to P100 latency, the P-value was not significant (Table 6).

**Table 6:** Regression analysis. *P-value less than 0.01 is considered statistically significant. ^HBA1C - Glycated hemoglobin, @Serum AGEs - Serum advanced glycated end products.

	Beta	P-value	95% Confidence Interval of Beta	
			Lower Bound	Upper Bound
With P100 latency recorded at baseline (24-28 weeks) as the dependent variable				
Mode of treatment	-0.60	0.74	-5.18	3.98
HBA1C^	6.10	0.02	-1.85	14.06
Serum AGEs^@^	0.05	0.13	-0.75	0.19

## Discussion

**Figure 1:**
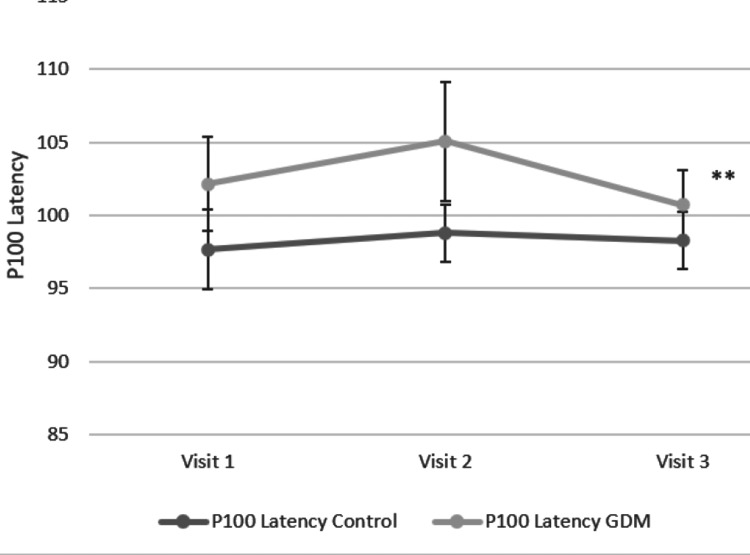
Trend lines showing the change in P100 latency of the left eye of the control and GDM groups across three visits during and after pregnancy (visit 1 - at 24-28 weeks of gestation, visit 2 - 32-36 weeks of gestation, visit 3 - 6-12 weeks after parturition). ** P-value for the difference of the mean P100 latencies within the GDM group among all three visits was less than 0.01. Error bars denote the standard deviation for the mean P100 latency values.

In our study, the observed P100 latency in the GDM group was significantly prolonged as compared to the control group at baseline, late pregnancy, and during postpartum visits. There was a significant drop in the mean P100 latency during postpartum visits in the GDM group as compared to late pregnancy, yet the value was still higher than that in the control group (Figure 1).

The N75-P100 amplitude was significantly increased in GDM at baseline but was not statistically different during the other two visits. The mean P100 latency in the GDM group at baseline and during late pregnancy was higher than the mean P100 latency reported for healthy pregnant females in our population [17]. The mean P100 latency in the GDM group during the postpartum visit was within the normal range, suggesting healthy females in our population [18].

Waves generated in VEP signify the summated postsynaptic potential of neurons involved in the processing of visual stimuli generated at the level of the striate cortex [19]. P100 wave is the most studied wave in VEP as it shows the least intra- and interindividual variations [19]. Conduction delay or nerve fiber injury anywhere along the visual pathway can be manifested by a change in latency or amplitude of the P100 wave [7]. Observations in our study suggest that short-term hyperglycemia of GDM was associated with an increase in P100 latency and a decrease in N75-P100 amplitude of VEP during pregnancy. These changes were consistent with results obtained in a previous study [14]. Prolonged P100 latency is reported in DM cases both with and without retinopathy [19]. Past studies have reported prolonged P100 latency in type 1 DM cases as soon as less than six months after diagnosis [9,10]. In a study by Schneck et al., acute alterations in blood glucose levels in diabetic patients without retinopathy were directly correlated to changes in latency in chromatic VEP [20]. More clinical research is needed to ascertain the exact relationship of short-term hyperglycemia with the degree of change in P100 latency [19]. An experimental study involving diabetic rat models has demonstrated that myelin sheath irregularities, along with functional loss of oligodendrocytes, astrocytes, and retinal ganglionic cells, were found in rats whose diabetes duration was six weeks [21].

In this study, the change in P100 latency was more consistent as compared to the change in amplitude from baseline recording to late pregnancy. Many studies have found P100 latency abnormalities than P100 amplitude abnormalities in DM cases, pointing to the fact that visual pathway involvement in diabetes could be due to conduction abnormalities at the myelin sheath level rather than ischaemic damage to the axons that affects the amplitude [7,19]. Prolonged P100 latency at baseline in our study was positively correlated with biochemical markers of the HBA1C and N-CML (a form of serum AGE) levels. Both HBA1C and AGE signify the degree of hyperglycemia, reflecting that prolonged P100 latency in GDM was directly related to the degree of hyperglycemia during pregnancy. This is consistent with a previous study reporting a positive correlation of P100 latency in GDM with the glycemic status of subjects [14]. A study by Korkmaz et al. reported prolonged P100 latency in glucose intolerant or pre-diabetic subjects as compared to normoglycemic controls, reflecting that P100 latency can be affected by milder forms of hyperglycemia than well-established diabetes [22]. Within the GDM group, women receiving insulin therapy reported longer mean P100 latencies during baseline and late pregnancy than women placed on diet therapy. As women with higher glycaemic status are placed under insulin treatment during pregnancy [15], this can also reflect the fact that the glycemic status can influence P100 latency even in a short duration of GDM.

The exact mechanism of alteration in VEP in GDM cases during pregnancy is not known. In our study, increased accumulation of AGEs in GDM was positively correlated with prolonged P100 latency during pregnancy. Sisay et al. in their meta-analysis found that GDM is associated with a significant accumulation of AGEs during pregnancy as compared to that of normoglycemic control [23]. AGE accumulation in the brain can increase the permeability of the blood-brain barrier (BBB) by forming cross-links with proteins such as collagen and acting on receptors of endothelial cells to induce their proliferation with the release of inflammatory cytokines [24]. The change in the integrity of BBB leads to the accumulation of several harmful products such as reactive oxygen species [24]. AGE accumulation in the brain also creates an environment of increased inflammation and oxidative stress by acting through RAGE [24]. Such changes have the potential to induce demyelinating damages in the visual pathway, causing prolonged P100 latency [25]. Increased AGE levels increase the expression of vascular endothelial growth factor (VEGF) in retinal endothelial cells [26]. VEGF increases the permeability of retinal blood vessels and causes the release of inflammatory cytokines, inducing metabolic changes in the retinal environment that can be manifested as prolonged P100 latency [25]. More studies are required to ascertain the exact pathophysiological mechanism of change in VEP during GDM.

Postpartum P100 latency in the GDM group was significantly reduced as compared to one recorded during late pregnancy. This can be due to improvement in glycemic status after removal of hormonal influences involved in increasing IR during pregnancy [27]. Improved postpartum P100 latency in the GDM group was still higher than the P100 latency obtained in the normoglycemic control group. The GDM group also had higher levels of AGE, HOMA IR, and HBA1C during postpartum visits as compared to controls. These changes are similar to previous studies reporting increased IR, beta-cell dysfunction, dyslipidemia, and increased AGE accumulation in GDM cases after delivery [28]. All of these factors predispose to increased incidence of cardiometabolic diseases in women with a history of GDM and risk of future DR [28]. Pregnancy in diabetes is considered an independent risk factor for DR [29]. Clinical guidelines suggest regular ophthalmoscopic screening for DR in pregnancies complicated by type 2 DM with postpartum follow-up [29]. However, no clearly defined guidelines exist for screening GDM cases for DR [29]. Recent evidence suggests metabolic memory of GDM poses a threat of microvascular retinal damage and subsequent DR in a greater percentage of women after pregnancy [5]. A study by Li et al., exploring the microvascular architecture of the retina in GDM cases at 26-28 weeks of gestation, revealed that retinal vascular changes, including narrower caliber, reduced fractal dimension, and increased branching angle points, were found as the onset of retinal vascular dysfunction in GDM. VEP can provide a window for the detection of small structural and metabolic changes that remain undetectable in ophthalmoscopy for the identification of at-risk patients for follow-up [30].

Our study attempted to record alterations in VEP associated with GDM during and after pregnancy and its relationship with metabolic dysfunction. It was limited by the short duration of postpartum follow-up due to time constraints. Longer longitudinal cohort studies are required to record the relationship of VEP alterations with the incidence of DR after delivery and its relationship with various biochemical markers of metabolic stress. VEP is limited by a large degree of variations across different subsets of populations [19]. Factors such as BMI, head circumference, age, height, illuminance, electrode placement, visual acuity, stimulation rate, type of checkerboard pattern, checkerboard size and contrast, and distance of the subject from the screen can affect the results in a VEP test [16]. While comparing different studies measuring VEP in DM, the influence of such factors should be kept in mind. Studies following uniform guidelines of VEP measurement in a single population should be compared. We have compared our results with studies following ISCEV guidelines while recording VEP in the same population as ours.

## Conclusions

Our study findings reflect that the diagnosis of GDM is associated with significant changes in VEP during and after pregnancy, as compared to healthy pregnant women. The change in P100 latency in the GDM group was more consistent as compared to the change in N75-P100 amplitude across pregnancy till postpartum follow-up. Although the postpartum P100 latency saw a significant drop in the GDM group as compared to late pregnancy, the values were still significantly higher than the healthy controls, reflecting that diagnosis of GDM can alter the normal electrophysiological functioning of the visual pathway even after pregnancy. With an increasing incidence of DR after pregnancy in the GDM population, VEP can provide a non-invasive tool for screening and follow-up of at-risk patients. The exact mechanism of the effect of GDM on visual pathway functioning is not known. Prolonged P100 latency in the GDM group during 24-28 weeks of gestation was significantly correlated to the accumulation of serum AGE levels. AGEs can alter the integrity of BBB along with predisposing to increased inflammation and oxidative stress that can alter signal conduction of visual pathways. Longer longitudinal cohort studies are required to record the relationship of VEP alterations with the incidence of DR after delivery and its relationship with various biochemical markers of metabolic stress.
